# Proton pump inhibitors enhance the effects of cytotoxic agents in chemoresistant epithelial ovarian carcinoma

**DOI:** 10.18632/oncotarget.5319

**Published:** 2015-09-19

**Authors:** Yoo-Young Lee, Hye-Kyung Jeon, Ji Eun Hong, Young Jae Cho, Ji Yoon Ryu, Jung-Joo Choi, Sang Hoon Lee, Gun Yoon, Woo Young Kim, In-Gu Do, Min Kyu Kim, Tae-Joong Kim, Chel Hun Choi, Jeong-Won Lee, Duk-Soo Bae, Byoung-Gie Kim

**Affiliations:** ^1^ Department of Obstetrics and Gynecology, Samsung Medical Center, Sungkyunkwan University School of Medicine, Seoul, Korea; ^2^ Department of Obstetrics and Gynecology, Chung-Ang University School of Medicine, Seoul, Korea; ^3^ Department of Obstetrics and Gynecology, Pusan National University Yangsan Hospital, Pusan National University School of Medicine, Yangsan, Korea; ^4^ Department of Obstetrics and Gynecology, Kangbuk Samsung Hospital, Sungkyunkwan University School of Medicine, Seoul, Korea; ^5^ Pathology, Kangbuk Samsung Hospital, Sungkyunkwan University School of Medicine, Seoul, Korea; ^6^ Department of Obstetrics and Gynecology, Samsung Changwon Hospital, Sungkyunkwan University School of Medicine, Changwon, Korea

**Keywords:** epithelial ovarian cancer, microenvironment, V-ATPase, omeprazole, clear cell carcinoma

## Abstract

This study was designed to investigate whether proton pump inhibitors (PPI, V-ATPase blocker) could increase the effect of cytotoxic agents in chemoresistant epithelial ovarian cancer (EOC). Expression of V-ATPase protein was evaluated in patients with EOC using immunohistochemistry, and patient survival was compared based on expression of V-ATPase mRNA from a TCGA data set. *In vitro*, EOC cell lines were treated with chemotherapeutic agents with or without V-ATPase siRNA or PPI (omeprazole) pretreatment. Cell survival and apoptosis was assessed using MTT assay and ELISA, respectively. *In vivo* experiments were performed to confirm the synergistic effect with omeprazole and paclitaxel on tumor growth in orthotopic and patient-derived xenograft (PDX) mouse models. Expression of V-ATPase protein in ovarian cancer tissues was observed in 44 patients (44/59, 74.6%). Higher expression of V-ATPase mRNA was associated with poorer overall survival in TCGA data. Inhibition of V-ATPase by siRNA or omeprazole significantly increased cytotoxicity or apoptosis to paclitaxel in chemoresistant (HeyA8-MDR, SKOV3-TR) and clear cell carcinoma cells (ES-2, RMG-1), but not in chemosensitive cells (HeyA8, SKOV3ip1). Moreover, the combination of omeprazole and paclitaxel significantly decreased the total tumor weight compared with paclitaxel alone in a chemoresistant EOC animal model and a PDX model of clear cell carcinoma. However, this finding was not observed in chemosensitive EOC animal models. These results show that omeprazole pretreatment can increase the effect of chemotherapeutic agents in chemoresistant EOC and clear cell carcinoma via reduction of the acidic tumor microenvironment.

## INTRODUCTION

Epithelial ovarian cancer (EOC) is the leading cause of death among all gynecological cancers and the five-year survival rate is dismal at 11% for patients with stage IV and 23%–41% for patients with stage III EOC [1]. One of the major obstacles to overcoming this bleak prognosis is tumor chemoresistance that arises following treatment with taxane and platinum-based agents, which is the standard regimen of chemotherapy for post-surgery EOC [2].

Chemoresistance of cancer cells can occur by genetic or epigenetic mechanisms [3, 4] but the acidic tumor microenvironment has also been shown to increase the chemoresistance of solid tumors [3]. For example, one of the most distinctive features of tumor cells is their cytoplasmic alkalization and subsequent acidification of the tumor microenvironment [5, 6]. Recently, high-dose proton pump inhibitors (PPI), which inhibit acidic microenvironments by blocking the activity of vacuolar H^+^-ATPases (V-ATPases), have been shown to enhance the effect of chemotherapeutic agents on chemoresistant tumors developed in companion animals [7].

This study was designed to investigate whether PPI increase the cytotoxic effect of chemotherapy in chemoresistant EOC cells *in vitro* and *in vivo*.

## RESULTS

### Expression of V-ATPase in patients with EOC and its clinical significance

**Figure 1:**
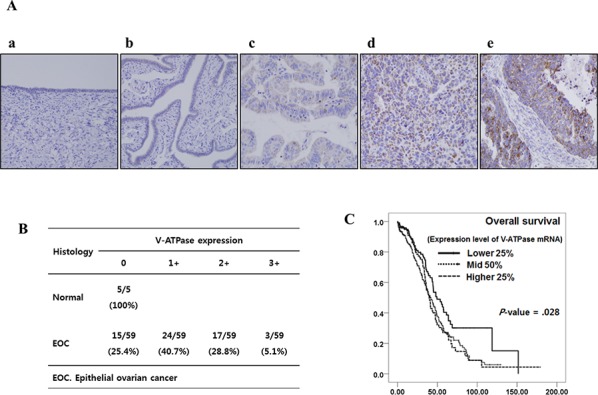
V-ATPase immunoreactivity in human ovarian epithelium and survival analysis based on mRNA expression of V-ATPase in patients with epithelial ovarian cancer (TCGA data) **A.** Representative V-ATPase staining from normal ovarian epithelium (a), and serous ovarian adenocarcinoma (b–d) with no staining (0), weak staining (+1), moderate staining (+2), strong staining (+3), respectively. (All photographs were taken at original 400x magnification) **B.** Distribution of patients with epithelial ovarian cancer according to the V-ATPase immunoreactivity **C.** Kaplan-Meier survival analysis showed higher overall survival in patients who showed lower 25% expression of mRNA expression.

Immunoreactivity of V-ATPase was predominantly observed in the cytoplasm of cells (Figure 1A). V-ATPase expression was not observed in any of the five normal ovarian and fallopian tube epithelium but was expressed in 44 of 59 EOC tissues (74.6%) as shown in Figure 1B. To investigate the clinical significance of V-ATPase expression in EOC, we used TCGA data and found that patients with higher 75 percentile mRNA expression of V-ATPase showed significantly poorer overall survival than patients with lower 25 percentile mRNA expression of V-ATPase (Figure 1C, HR; 1.493, 95% CI; 1.109–2.009).

### V-ATPase siRNA transfection significantly increases the cytotoxicity of paclitaxel in chemoresistant cells

**Figure 2:**
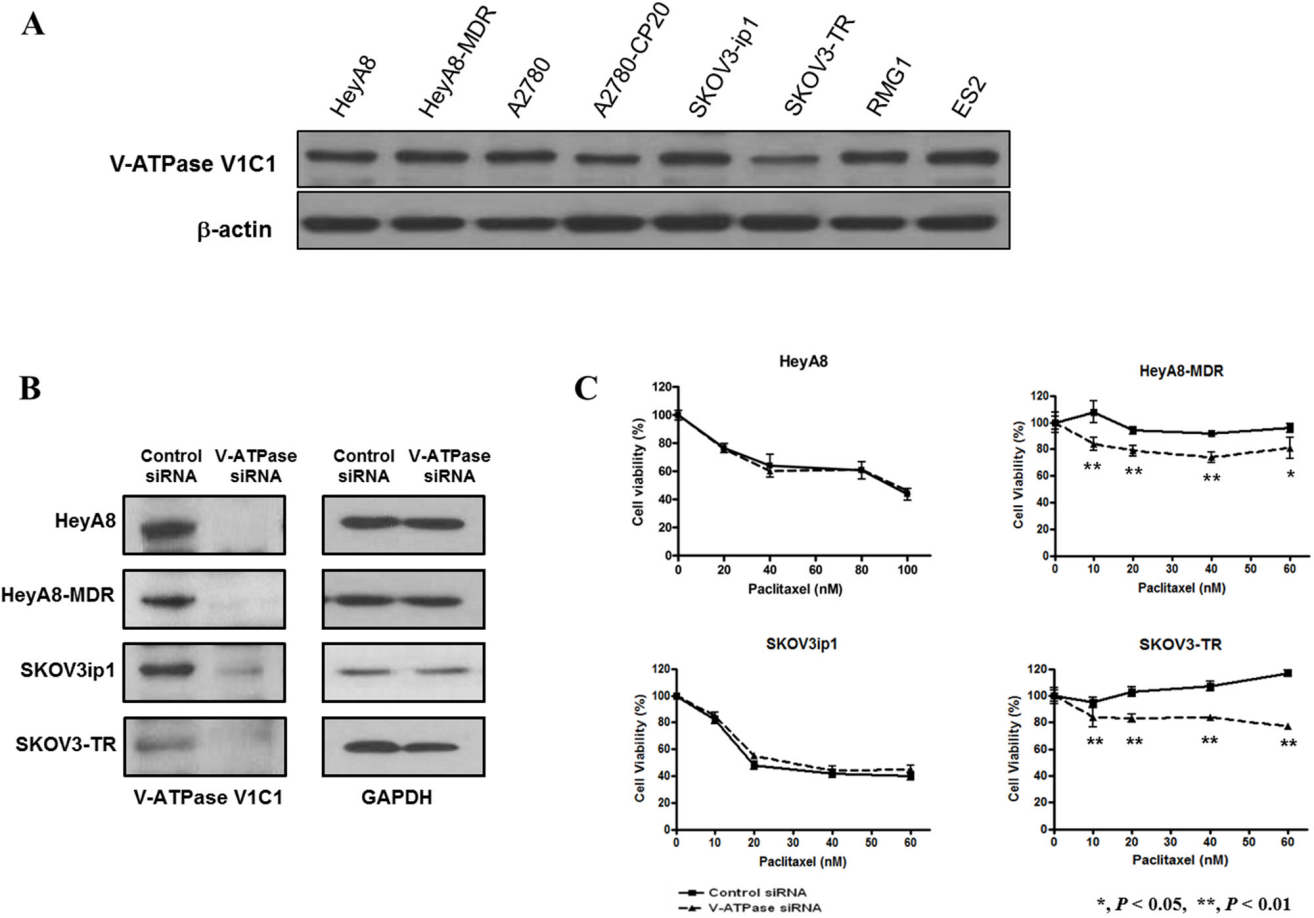
Western blot analysis for protein expression of V-ATPase in epithelial ovarian cancer cell lines and the effects of V-ATPase specific siRNA on cytotoxicity of paclitaxel in epithelial ovarian cancer cell lines **A.** Variable expression of V-ATPase V1C1 was observed in epithelial ovarian cancer cell lines. **B.** Knockdown of V-ATPase expression siRNA transfection assessed by Western blot analysis in epithelial ovarian cancer cell lines. **C.** Cell survival significantly decreased in V-ATPase siRNA and paclitaxel-treated cells compared with paclitaxel alone in chemoresistant cell lines. (HeyA8, SKOV3ip1, and A2780-PAR; chemosensitive cell lines, HeyA8-MDR, SKOV3-TR, and A2780-CP20; chemoresistant cell lines). Bar, standard deviation.

V-ATPase protein expression was initially assessed in several EOC cell lines. Western blot analysis showed variable expression of V-ATPase protein in EOC cell lines (Figure 2A). In order to test whether inhibition of V-ATPase could enhance chemosensitivity, EOC cell lines were transfected with V-ATPase specific siRNA (Figure 2B and 2C). Inhibition of V-ATPase significantly increased cytotoxicity to paclitaxel in HeyA8-MDR (20%, *P* < 0.05) and SKOV3-TR cells (20%, *P* < 0.05), but not in chemosensitive cell lines HeyA8 and SKOV3ip1. When we extended exposure time of the cytotoxic drugs to 72 and 96 hours, respectively, the results were the same (supplementary Figure 1A).

### Intracellular pH decreases after PPI treatment

**Figure 3:**
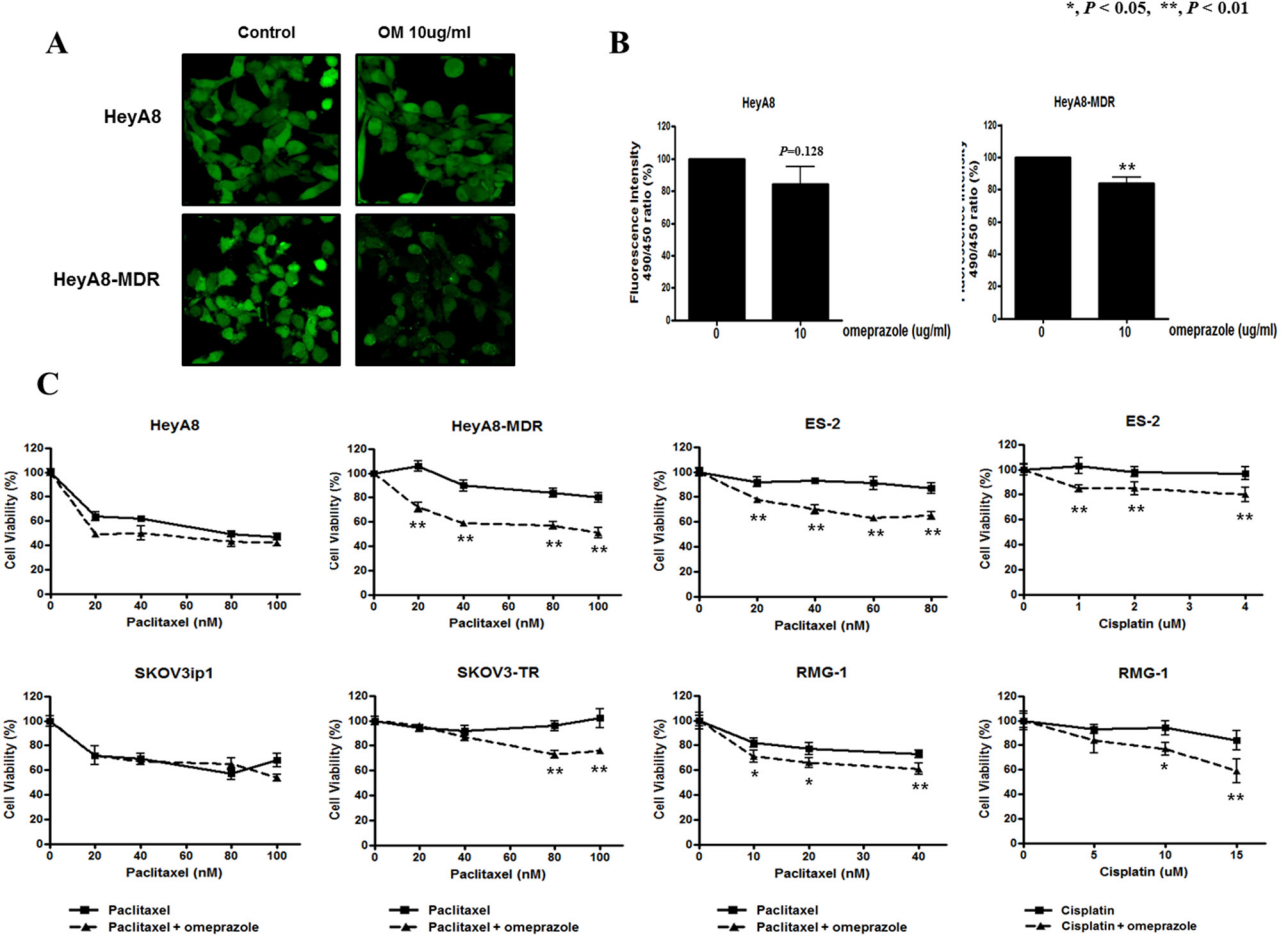
Measurement of pH after omeprazole treatment and effects of omeprazole on cell survival with cytotoxic drugs in epithelial ovarian cancer cell lines **A, B.** Significantly decreased intracellular pH was observed in omeprazole (20 mg/mL) treated chemoresistant cell lines (HeyA8-MDR). **C.** Omeprazole pretreatment was significantly associated with decreased cell viability measured by MTT assay in chemoresistant cell lines (HeyA8-MDR, ES-2, RMG-1). Bar, standard deviation.

To confirm the change of pH in cells by PPI treatment, alterations in intracellular pH in HeyA8 and HeyA8-MDR cells were verified using the BCECF-AM pH indicator. In chemoresistant HeyA8-MDR cells, fluorescence significantly decreased, indicating that intracellular pH was acidified by V-ATPase inhibition. In contrast, intracellular pH showed no significant change in chemosensitive HeyA8 cells (Figure 3A). Additionally, quantitative analysis showed that intracellular pH decreased with statistical significance in HeyA8-MDR cells, but statistical significance was not achieved in HeyA8 cells (Figure 3B).

### PPI pretreatment significantly increases the cytotoxicity and apoptosis of chemotherapeutic agent in chemoresistant EOC cells

We then assessed whether pretreatment with omeprazole could reverse the sensitivity to chemotherapy in chemoresistant cell lines (taxane-resistant including HeyA8-MDR and SKOV3-TR; clear cell carcinoma cell lines including ES-2 and RMG-1). The results showed that pretreatment with omeprazole significantly decreased cell survival after paclitaxel treatment in HeyA8-MDR cells by > 30% (*P* < 0.05) when compared to treatment with paclitaxel alone. However, this finding was not observed in HeyA8 cells, which are sensitive to paclitaxel (Figure 3C). When we extended exposure time of the cytotoxic drugs to 72 and 96 hours, respectively, the results were the same (supplementary Figure 1B). Similar results were obtained with SKOV3-TR (30%, *P* < 0.05) and SKOV3ip1 (no difference). Clinically, clear cell histology among EOC has poorer prognosis than other EOC subtypes due to its resistance to chemotherapy [8, 9]. Experiments conducted using clear cell carcinoma cell lines including ES-2 and RMG-1 showed that pretreatment with omeprazole could increase the cytotoxicity to paclitaxel and cisplatin compared with drug alone (Figure 3C).

**Figure 4:**
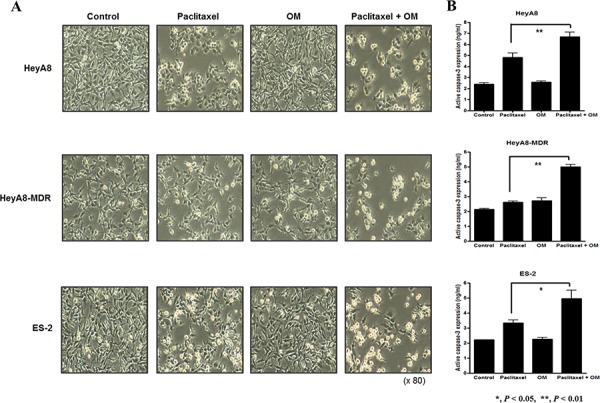
Involvement of omeprazole in cytotoxic drug-induced cell death **A.** Cell death was observed by light microscopy in each cell line (HeyA8, HeyA8-MDR, and ES-2). **B.** Expression of active caspase-3, measured by ELISA, was significantly increased in the omeprazole and paclitaxel group compared to paclitaxel alone. Bar, standard deviation. (OM; omeprazole)

To assess cell apoptosis, active caspase-3 was measured by ELISA in EOC cells HeyA8, HeyA8-MDR, and ES-2 treated with paclitaxel, with or without omeprazole pretreatment. Omeprazole pretreatment significantly increased the apoptotic activity of chemotherapy in all three cell lines and, interestingly, the increase in apoptosis with omeprazole was larger in chemoresistant cell lines HeyA8-MDR and ES-2 than in the chemosensitive cell line HeyA8 (Figure 4).

### PPI pretreatment significantly decreases tumor growth in chemoresistant cell line orthotopic xenografts of EOC

**Figure 5:**
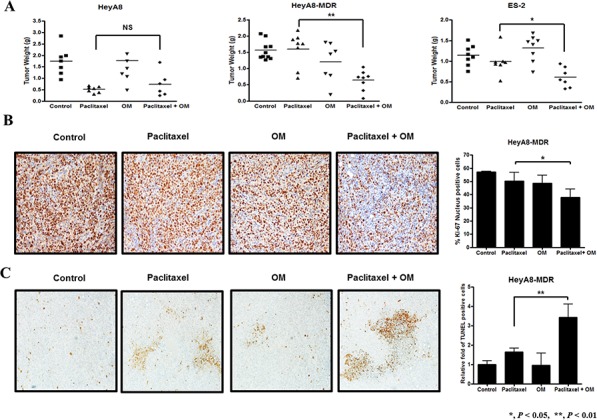
The effect of omeprazole as a chemosensitizer in chemoresistant cells assessed in female BALB/c nude mice **A.** Tumor weights were only significantly lower in chemoresistant animal models (HeyA8-MDR, ES-2) treated with omeprazole and paclitaxel compared to paclitaxel alone. **B.** Tumor cell proliferation, assessed by Ki-67 immunohistochemistry staining, in harvested tumor tissues was significantly lower in the omeprazole and paclitaxel group compared with paclitaxel alone. **C.** Apoptotic activity in harvested tumor tissues was significantly higher in the omeprazole and paclitaxel group (paclitaxel; 120 μg/mouse, OM; omeprazole; 50 mg/kg, NS; not significant). Columns, mean of 10 mice. Bars, standard error. (All photographs were taken at original magnification × 100)

In order to assess the potential clinical relevance of the *in vitro* results, *in vivo* experiments were performed using an EOC orthotopic animal model. HeyA8, HeyA8-MDR, or ES-2 cells were injected into the abdominal cavity of athymic nude mice. Mice injected with HeyA8-MDR or ES-2 cells and treated with paclitaxel in combination with pretreated omeprazole had significantly decreased tumor weight compared with those mice treated with paclitaxel alone (*P* < 0.01 and *P* < 0.05, respectively, Figure 5A). However, this finding was not observed in mice injected with chemosensitive HeyA8 cells, which is consistent with the results of our *in vitro* studies. In order to validate the results of our *in vitro* PPI pretreatment studies, we used mouse tumor tissue to assess for cell proliferation and apoptosis. Cell proliferation was evaluated using immunohistochemistry staining for Ki-67, which revealed that paclitaxel with omeprazole pretreatment significantly decreased the number of proliferating cells compared with paclitaxel alone (*P* < 0.05, Figure 5B). Additionally, paclitaxel with omeprazole significantly increased apoptosis, assessed by TUNEL assay, compared with paclitaxel alone (*P* < 0.01, Figure 5C).

### PPI pretreatment significantly decreases the tumor growth in a PDX model of clear cell carcinoma

**Figure 6:**
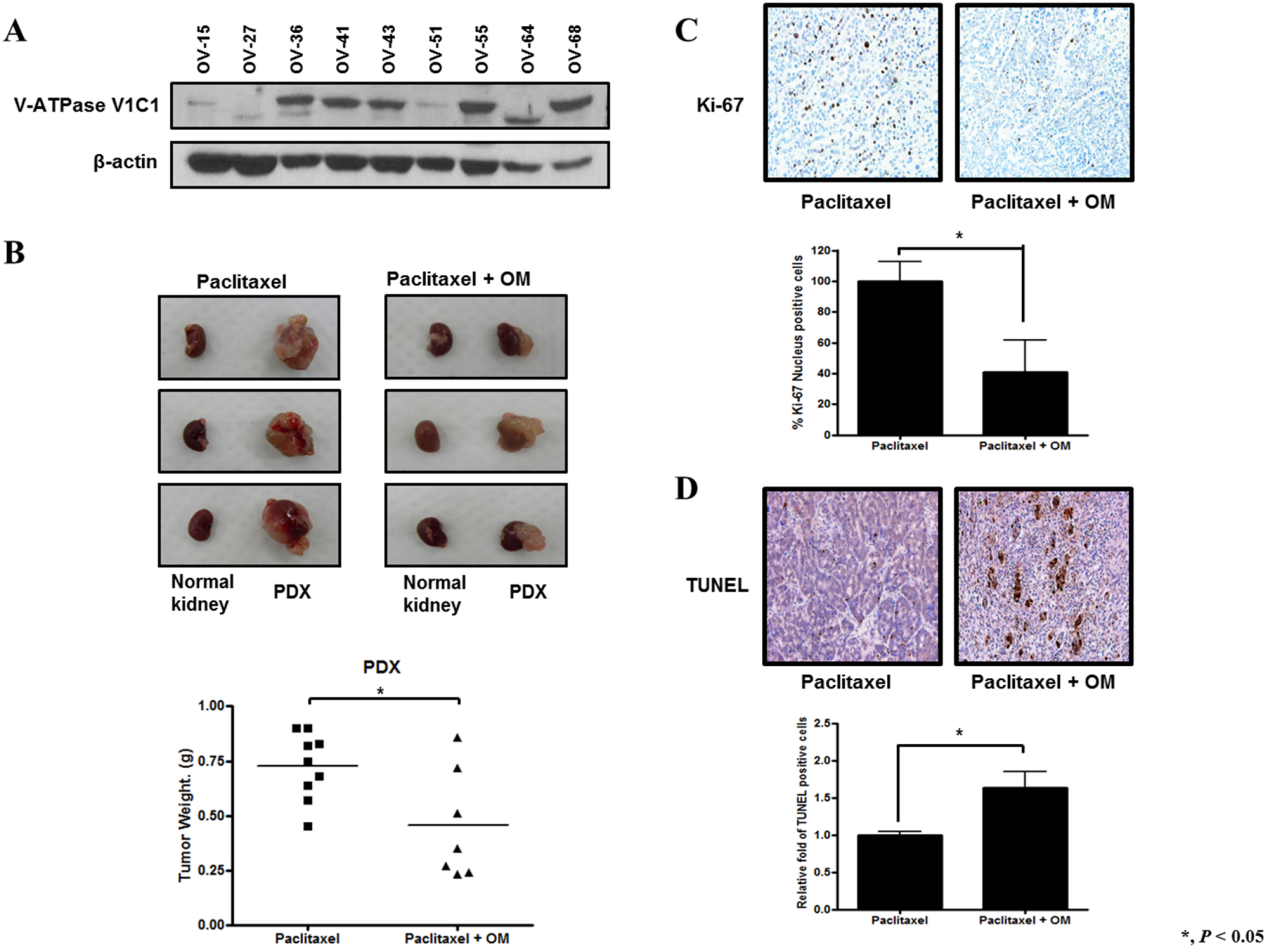
Effects of omeprazole on paclitaxel in a patient-derived tumor xenograft (PDX) model of platinum resistant ovarian clear cell carcinoma (ov-68) **A.** V-ATPaseV1C1 protein expression was variable in PDX models of EOC. **B.** Omeprazole resulted in a significantly decreased tumor weight compared with paclitaxel injected control. In each picture, the small left piece is normal kidney (no tumor transplanted), and the right large piece is developed PDX. **C.** Tumor cell proliferation with Ki-67 immunohistochemistry in harvested tumor tissues was significantly decreased in the omeprazole treated group. **D.** Apoptotic activity in harvested tumor tissues was significantly increased in the omeprazole and paclitaxel group. Bar, standard error.

We developed a PDX model of clear cell carcinoma using subrenal implantation of human ovarian cancer tissue. We assessed V-ATPase protein expression in several models of PDX of EOC and found various expression levels (Figure 6A). We selected case number OV-68, a clear cell carcinoma with relatively high expression of V-ATPase, for an *in vivo* therapy experiment. The patient from which this model was derived was 44 years old at the time of her initial visit and presented with FIGO stage IIIA ovarian cancer with clear cell carcinoma. The ovarian cancer tissues obtained during initial surgery were used for this model. The patient received six cycles of paclitaxel and carboplatin after optimal debulking surgery but had recurrent disease six months after the last dose of chemotherapy, which is clinically platinum resistant. The PDX model was treated for 3 weeks, beginning 5 weeks after implantation of xenograft tissues (passage 3). Omeprazole pretreatment significantly inhibited tumor growth compared with paclitaxel alone in this model (*P* < 0.05) (Figure 6B). Pretreatment with omeprazole also inhibited cell proliferation and increased apoptosis in this model (Figure 6C and 6D, respectively).

## DISCUSSION

In examining epithelial ovarian cancer patients in a TCGA cohort, we found that higher expression of V-ATPase mRNA was significantly associated with poor survival. In our *in vitro* studies, V-ATPase expression significantly decreased after EOC cell lines were treated with specific inhibitors, including siRNA and omeprazole, and pretreatment with omeprazole to inhibit V-ATPase function sensitized chemoresistant cells to paclitaxel treatment. In an *in vivo* orthotopic chemoresistant mouse model of EOC, tumor volume significantly decreased following chemotherapy only when omeprazole was used for pretreatment. These results were corroborated in a PDX animal model derived from a patient whose clear cell carcinoma of the ovary showed platinum resistance, in which pretreatment with omeprazole significantly reduced tumor growth compared to paclitaxel alone.

V-ATPases are ATP dependent H^+^ transporters, which regulate intra- and extracellular pH by actively transporting protons [10]. For tumor cells, cytoplasmic acidification may be lethal because this acidification can trigger a cascade of lytic enzymes that ultimately lead to self-digestion [11]. V-ATPases in tumor cells thus maintains an appropriate relatively neutral intracellular pH and an acidic extracellular pH [12]. As a result, the acidic microenvironment increased by overexpression of V-ATPases is observed in malignant tissues [13]. For example, higher expression of V-ATPases in cancer cells is associated with poorer prognosis [14], and knockdown of V-ATPase expression by siRNA in a human hepatocellular carcinoma was found to markedly slow the growth rate and block the metastatic spread in nude mice [15].

Proton pump inhibitors such as omeprazole, used clinically to suppress gastric acidity in gastritis, are activated by acidic conditions and tend to decrease intracellular pH and increase the extracellular pH via inhibition of V-ATPases in a covalent interaction [16]. The pharmacodynamics of PPIs has been shown to have anti-proliferative and pro-apoptotic effects on certain cancer cell lines [11, 17–20]. Clinically, omeprazole is safe for treating gastritis and esophagitis, but it is frequently associated with drug interactions. Omeprazole has high affinity for cytochrome P450 (CYP) enzymes and is primarily metabolized by this enzyme. It has therefore been suggested that omeprazole might stimulate the effect of anti-cancer agents which are also metabolized by CYP enzyme. However, until now, reports studying drug interactions have not found that omeprazole alters the pharmacokinetics and toxicities of these anti-cancer agents. With the safety and convenient administration of omeprazole, there are potential benefits in clinical application.

Among the histotypes of EOC, clear cell ovarian carcinoma is known for its distinctive histopathological and molecular genetic features, including chemoresistance [21, 22]. In this study, clear cell carcinoma cell lines showed chemoresistance, which was abrogated by blocking V-ATPase via omeprazole. In addition, in a PDX animal model derived from a patient with chemoresistant clear cell ovarian cancer, there was higher expression of V-ATPase in tumor tissue and poor tumor response to paclitaxel alone; however, omeprazole pretreatment increased the sensitivity of the tumor to paclitaxel. These findings suggest V-ATPase as a candidate target molecule or biomarker for cancer treatment and provide a potential role for omeprazole as a chemosensitizer in clear cell carcinoma of the ovary. Clinical trials with this regimen should be seriously considered in the future, especially considering that no chemotherapeutic agents have demonstrated efficacy for clear cell carcinoma of the ovary until now.

In conclusion, omeprazole enhanced the effect of chemotherapeutic agents on chemoresistant cells and animal models. This data suggests that omeprazole may be useful as a chemosensitizer in treatment of patients with chemoresistant EOC.

## MATERIALS AND METHODS

### Patients and tissue specimens

Fifty-nine patients who underwent surgery for EOC at the Department of Obstetrics and Gynecology at Samsung Medical Center between October 2003 and November 2005 were included in this study. Five normal ovarian and fallopian tube specimens were used as normal controls. Tumor tissue specimens were obtained during surgery from women with epithelial ovarian carcinoma. A single gynecologic pathologist (I-G Do) examined the specimens using hematoxylin and eosin staining. Specimens were used in analysis if they comprised more than 90% tumor cells. This study was reviewed and approved by the Institutional Review Board at Samsung Medical Center, Seoul, Korea (IRB No. 2011-04-008-002). We also investigated the prognostic role of ATP6V1C1 gene expression using unprotected data of 489 patients from The Cancer Genome Atlas (TCGA) [23].

### Immunohistochemical analysis

Immunohistochemical studies were carried out on formalin-fixed, paraffin-embedded, 4 μm thick tissue sections. The primary antibodies used were rabbit polyclonal V-ATPase subunit C1 antibody (Santa Cruz Biotechnology, Santa Cruz, CA) and anti-Ki-67 (DAKO, Glostrup, Denmark). Tissue sections were deparaffinized three times in xylene for a total of 15 minutes and subsequently rehydrated. Immunostaining for V-ATPase was performed using a Bond-max^TM^ automated immunostainer (Leica Biosystems, Melbourne, Australia) and the Bond^TM^ Polymer Refine Detection kit (Vision Biosystems, Melbourne, Australia). Briefly, antigen retrieval was carried out at 97°C for 20 minutes in ER1 buffer. After blocking endogenous peroxidase activity with 3% hydrogen peroxidase for 10 minutes, primary antibody incubation was carried out for 15 minutes at room temperature at a dilution of 1:200. Anti-mouse IgG (AI-2000, Vector Laboratories, Burlingame, CA) was used in place of primary antibody as a negative control. The intensity of staining was graded on a semiquantitative scale from 0 to 3, where 0 = no staining, 1+ = weak staining, 2+ = moderate staining, and 3+ = strong staining. Immunohistochemistry for Ki-67 was performed as described previously [24]. To quantify Ki-67 expression, the number of Ki-67 positive cells and the total number of tumor cells were counted in 5 random fields at 100x magnification and the percentage of positive cells was calculated.

### TUNEL assay

Apoptotic cell death was assessed using a terminal deoxynucleotidyl transferase-mediated dUTP nick end labeling (TUNEL) assay with a commercially available apoptosis detection kit (Promega, Fitchburg, WI) according to the manufacturer's instructions [25]. Briefly, after routine deparaffinization, rehydration, and blocking of endogenous peroxidase with 3% hydrogen peroxide in PBS for 10 min at room temperature, tissue sections were digested with 20 μg/mL proteinase K in PBS for 15 min at room temperature. After washing sections in PBS buffer, equilibration buffer was applied for 5 min at room temperature, and the sections were then incubated with working strength terminal deoxynucleotidyl transferase (TdT) enzyme for 60 min at 37°C in a humidity chamber. The reaction was terminated in working strength stop/wash buffer for 15 min at room temperature. After washing in PBS, the sections were covered with Streptavidin HRP solution for 30 min at room temperature, and the color reaction was developed using DAB substrate chromogen solution for 5 min and then washed with distilled water. Sections were lightly counterstained with Mayer's hematoxylin for 30 sec. All sections were also stained with H&E for histologic evaluation. To quantify cell death, the number of TUNEL-positive cells was counted in 5 random fields at 100x magnification and the percentage of positive cells was calculated.

### Cell lines

Human EOC cell lines (HeyA8, SKOV3ip1, HeyA8-MDR, SKOV3-TR, and A2780-CP20) were a gift from Dr. Anil K. Sood, Department of Cancer Biology, University of Texas M.D. Anderson Cancer Center, TX, USA [26]. A2780 and ES-2 cell lines were obtained from the American Type Culture Collection (ATCC, Manassas, VA). The RMG-1 cell line was obtained from Japan Health Science Research Resources Bank (HSRRB, Osaka, Japan). Human EOC cell lines were maintained in complete media (HeyA8, HeyA8-MDR, SKOV3ip1, SKOV3-TR, A2780, and A2780-CP20: RPMI 1640, ES-2: McCoy's 5A, RMG-1: Ham's F12) supplemented with 10% fetal bovine serum (FBS) and 0.1% gentamicin sulfate (Gemini Bioproducts, Calabasas, CA) in 5% CO_2_ at 37°C.

### Western blot analysis

Preparation of lysates from cultured cells and tumors has been described previously [27]. Protein bands were probed with V-ATPase subunit C1 antibody at 1:1000 dilution (Santa Cruz Biotechnology, Santa Cruz, CA) and tubulin antibody at 1:3000 dilution (Epitomics, Burlingame, CA), and then probed with horseradish peroxidase-conjugated anti-rabbit and anti-goat antibody (GE Healthcare, Piscataway, NJ). Bands were visualized by enhanced chemoluminescence using an ECL kit (Amersham Biosciences, Buckinghamshire, UK) according to the manufacturer's protocol.

### Small interfering RNA (siRNA) transfection and drug treatment

*V-ATPase V1C1* siRNA and negative control siRNA were obtained from Santa Cruz Biotechnology. Cells were seeded at 4 × 10^3^ cells/well in a 96-well microplate in RPMI 1640 with 10% FBS. All siRNAs (10 nM) were transfected into cells using Lipofectamine 2000 (Invitrogen, San Diego, CA) according to the manufacturer's protocol. After 24 h of siRNA transfection, cells were treated with various concentrations of paclitaxel (Sigma-Aldrich, St. Louis, MO) and then incubated at 37°C for 48, 72, and 96 h. Omeprazole (AstraZeneca, Mölndal, Sweden) was resuspended in normal saline at a concentration of 5 mg/mL. Cells were seeded at 4 × 10^3^ cells/well in a 96-well microplate in RPMI 1640 with 10% FBS. Cells were pretreated with or without omeprazole (20 mg/mL) based on previous reports [28], and after 24 h of treatment, cells were treated with various concentrations of paclitaxel and incubated at 37°C for 48, 72, and 96 h.

### 3-(4, 5-dimethylthiazol-2-yl)-2, 5-diphenyl tetrazolium bromide (MTT) assay

The MTT assay was performed as previously described [29]. Each sample was assayed in triplicate.

### Measurement of intracellular pH levels

BCECF-AM(2′,7′bis(2carboxyethyl)5(and6)carboxyfluorescein, (Invitrogen) is the most widely used fluorescent indicator for intracellular pH. The BCECF-AM was excited at 488 nm with emission collected at 500–550 nm [30]. HeyA8 and HeyA8-MDR (4 × 10^5^ cells) cells were seeded in a 35 mm confocal dish (#200350, SPL Lifescience, Pocheon, Gyeonggi, Korea) in RPMI 1640 with 10% FBS. The next day, cells were treated with omeprazole (10 μ g/mL) in serum free RPMI 1640 without sodium bicarbonate. After 24 h of treatment, the cells were treated with 1 μ g/mL BCECF-AM solution, incubated at 37°C for 30 minutes and analyzed by LSM 700 confocal microscope.

To measure 490 nm/450 nm ratio [31, 32], HeyA8 and HeyA8-MDR (2 × 10^4^ cells/well) cells were seeded in a 96-well black microplate in RPMI 1640 with 10% FBS. After two days, cells were treated with omeprazole (10 μg/mL) in serum free RPMI 1640 without sodium bicarbonate. After 24 h of treatment, the cells were treated with 1 μg/mL BCECF-AM solution, incubated at 37°C for 30 minutes and analyzed by PerkinElmer VICTOR2 plate reader. Data corrected to cell numbers by using MTT assay.

### Animal care and development of *in vivo* models, including established cell line xenografts and patient-derived xenografts (PDX)

Female BALB/c nude mice were purchased from Orient Bio (Seongnam, Korea). This study was reviewed and approved by the Institutional Animal Care and Use Committee (IACUC) of the Samsung Biomedical Research Institute (protocol No. H-A9–003), which is an Association for Assessment and Accreditation of Laboratory Animal Care International (AAALAC International) accredited facility and abides by the Institute of Laboratory Animal Resources (ILAR) guide. To establish orthotopic cell line xenografts, HeyA8 (0.25 × 10^6^ cells/0.2 mL HBSS), HeyA8-MDR (1.0 × 10^6^ cells/0.2 mL HBSS), and ES-2 (7.5 × 10^5^ cells/0.2 mL HBSS) were injected into the peritoneal cavity of mice [33]. For the PDX model of EOC, surgical patient tumor specimens were reduced into small pieces (less than 2–3 mm^3^), implanted into the subrenal capsule of the left kidney, and propagated by serial transplantation [34]. After 6 days of cell injection for the cell line models or 5 weeks for the PDX models, omeprazole (50 mg/kg) based on previous studies [28, 35] or PBS were pre-injected i.p. After 24 h paclitaxel (120 μg/mouse) or PBS were injected i.p. once weekly in 200 μl volume. Four groups of cell line xenograft mice (*n* = 10 per group) for each cell line and two groups for PDX (*n* = 10 per group) were monitored for adverse effects. Tumors were harvested after 4 weeks of therapy or when any of the mice began to appear moribund. Total body weight and tumor weight of each mouse were recorded. Tumors were fixed in formalin and embedded in paraffin or snap frozen in OCT compound (Sakura Finetek Japan, Tokyo, Japan) in liquid nitrogen.

### Statistical analysis

The Mann–Whitney *U* test was used to evaluate the significance and to compare differences among groups for *in vitro* and *in vivo* assays. Overall and progression-free survival curves were evaluated and compared according to the Kaplan-Meier method using the log-rank test. SPSS software (version 17.0; SPSS, Chicago, IL) was used for all statistical analyses. All *P*-values were two-sided and considered statistically significant if *P* < 0.05.

## SUPPLEMENTARY FIGURE


