# Supplementary material for: Proton pump inhibitors enhance the effects of cytotoxic agents in chemoresistant epithelial ovarian carcinoma
Source: Oncotarget. 2015 Sep 19;6(33):35040–50. doi: 10.18632/oncotarget.5319 (PMC4741507; doi:10.18632/oncotarget.5319)
Supplement: Supplementary file 1 [file oncotarget-06-35040-s001.pdf]

SUPPLEMENTARY FIGURE

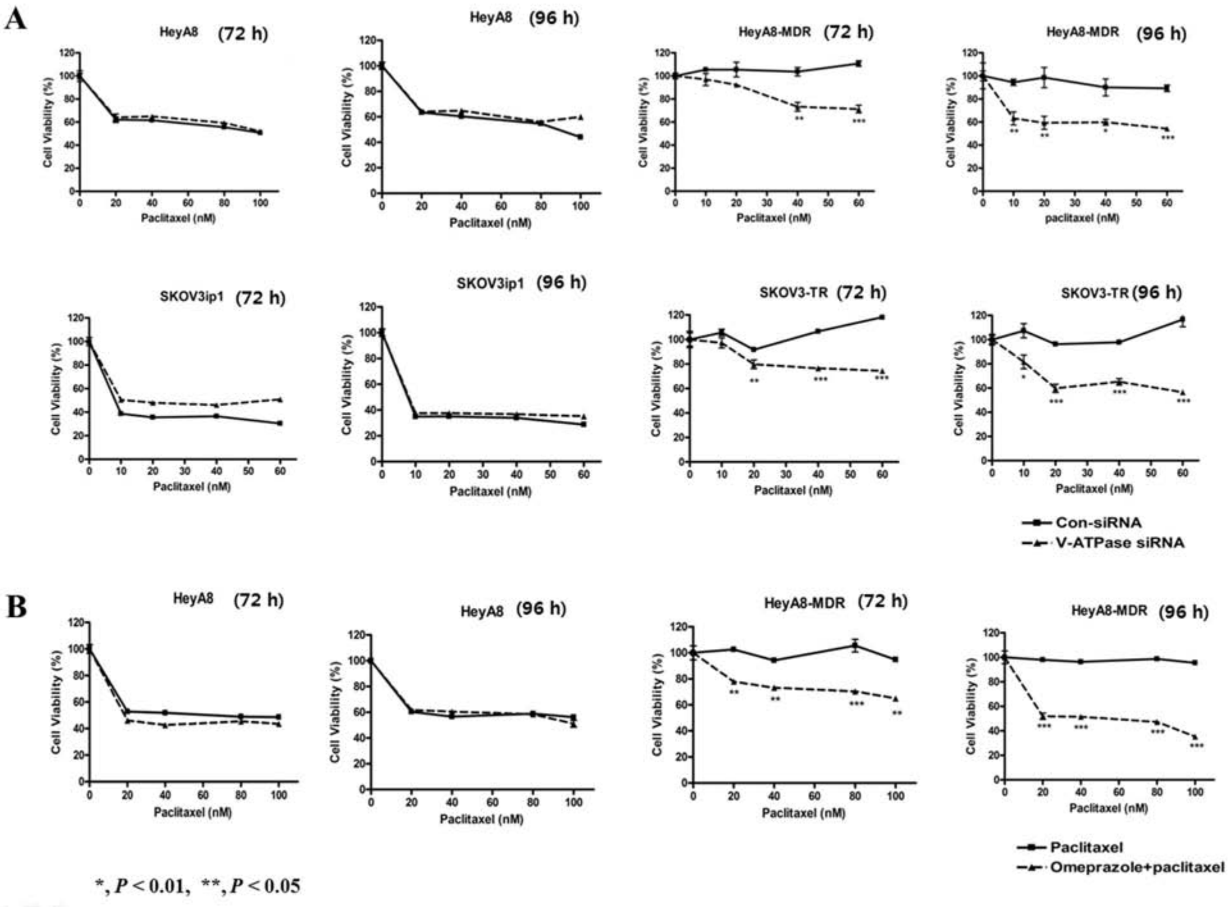

Supplementary Figure S1: The effects of V-ATPase specific siRNA A. and omeprazole B. on cytotoxicity of paclitaxel in epithelial ovarian cancer cell lines. Cell survival significantly decreased in V-ATPase siRNA and paclitaxel-treated cells compared with paclitaxel alone in chemoresistant cell lines. Omeprazole pretreatment was significantly associated with decreased cell viability measured by MTT assay in chemoresistant cell lines (HeyA8, SKOV3ip1; chemosensitive cell lines, HeyA8-MDR, SKOV3-TR; chemoresistant cell lines). Bar, standard deviation.
